# Analysing Tumour Growth Delay Data from Animal Irradiation Experiments with Deviations from the Prescribed Dose

**DOI:** 10.3390/cancers11091281

**Published:** 2019-08-31

**Authors:** Leonhard Karsch, Elke Beyreuther, Doreen Eger Passos, Jörg Pawelke, Steffen Löck

**Affiliations:** 1 Helmholtz-Zentrum Dresden—Rossendorf, Institute of Radiooncology—OncoRay, 01328 Dresden, Germany; 2 OncoRay—National Center for Radiation Research in Oncology, Faculty of Medicine and University Hospital Carl Gustav Carus, Technische Universität Dresden, Helmholtz-Zentrum Dresden—Rossendorf, 01328 Dresden, Germany; 3 Helmholtz-Zentrum Dresden—Rossendorf, Institute of Radiation Physics, 01328 Dresden, Germany; 4 Department of Radiotherapy and Radiation Oncology, Faculty of Medicine and University Hospital Carl Gustav Carus, Technische Universität Dresden, D-01062 Dresden, Germany; 5 German Cancer Consortium (DKTK), Partner Site Dresden, and German Cancer Research Center (DKFZ), 69120 Heidelberg, Germany

**Keywords:** pre-clinical studies, statistical analysis, experimental beams, radiotherapy

## Abstract

The development of new radiotherapy technologies is a long-term process, which requires proof of the general concept. However, clinical requirements with respect to beam quality and controlled dose delivery may not yet be fulfilled. Exemplarily, the necessary radiobiological experiments with laser-accelerated electrons are challenged by fluctuating beam intensities. Based on tumour-growth data and dose values obtained in an in vivo trial comparing the biological efficacy of laser-driven and conventional clinical Linac electrons, different statistical approaches for analysis were compared. In addition to the classical averaging per dose point, which excludes animals with high dose deviations, multivariable linear regression, Cox regression and a Monte-Carlo-based approach were tested as alternatives that include all animals in statistical analysis. The four methods were compared based on experimental and simulated data. All applied statistical approaches revealed a comparable radiobiological efficacy of laser-driven and conventional Linac electrons, confirming the experimental conclusion. In the simulation study, significant differences in dose response were detected by all methods except for the conventional method, which showed the lowest power. Thereby, the alternative statistical approaches may allow for reducing the total number of required animals in future pre-clinical trials.

## 1. Introduction

The development of new radiotherapy (RT) beam delivery techniques, e.g., laser driven particle acceleration [1], micro beam RT [2] or ultra-high dose rate irradiation (FLASH) [3], is a long-term process, where the general concept should be proven early on, even though clinical requirements, such as a stable dose delivery, are not yet fulfilled. In particular, with regard to the later application in RT, the respective concepts should be tested in a translational manner [4] to validate their ability of tumour killing and the effects on the surrounding normal tissue. Starting with physical optimization and in vitro experiments, a successful concept will be validated by in vivo trials before considering it for clinical application. Regarding the requirements of stability and reproducibility of beam parameters, animal trials are more challenging than in vitro experiments, since these trials cannot be prolonged or repeated easily for ethical reasons. 

Deviations from the prescribed dose may occur in all experiments, where pulsed radiation is applied. Compared to continuous beam delivery, the pulsed mode is most often harder to control and might result in over- or under-dosage. Another much less likely scenario is the failure of dosimetric equipment or an accidental change of beam parameters, e.g., by using wrong settings of beam current, beam filtration or treatment distance. For the statistical analysis of the corresponding data, it is important to apply methods that allow for the inclusion of data points with deviations in dose delivery. These methods achieve a higher power for detecting differences between differing treatments, which is of particular importance for animal trials with their limited number of individuals and the current aim to reduce animal numbers. 

One particular example, for which a stable dose delivery is challenging, is the concept of laser-driven particle acceleration. Its aim was to provide compact proton and ion accelerators that might fit into existing hospitals [5,6] and replace the large particle accelerators. Due to the acceleration by means of high-intensity lasers [6] the particle beams produced by this new technique are characterized by very short (picosecond) beam pulses of very high pulse dose rates in the order of 10^10^ Gy/s. In comparison, clinical RT typically delivers dose rates of up to about 10^3^ Gy/s. Consequently, the radiobiological properties of these new particle beams have to be investigated before any clinical application. 

Starting more than one decade ago, the first in vitro experiments with laser-driven electrons [7] and protons [8,9,10] were followed by two in vivo trials [11,12] aiming on the biological effects of pulsed laser-driven electron beams. In order to compare the radiobiological effect of laser-driven and conventional clinical Linac electrons, the tumour growth time after treatment with two different dose groups was evaluated at both accelerators [12]. While the dose delivery of the clinical Linac was stable and reproducible, leading to a maximum deviation from the prescribed dose of about 2%, beam-intensity fluctuations of the laser-accelerated electrons were not fully compensated by online dosimetry, which resulted in deviations of more than 10 % from the prescribed dose as measured by retrospective absolute film dosimetry. The common methods for evaluating tumour-growth data, however, are based on grouping of animals treated with a similar dose (e.g., [13,14,15,16]). Consequently, 20 of 47 animals treated with laser-driven electrons were excluded from analysis, reducing the statistical power to detect significant differences between the beam qualities. 

On the basis of the data derived by Oppelt et al. [12], the present work describes and compares different statistical approaches with the goal to maximize the number of animals in the analysis of in vivo trials by including animals with deviations from the prescribed dose. Methods feasible with commercial statistical software, like multivariable linear regression and Cox regression, but also a Monte-Carlo-based approach were applied to the experimental dataset. In addition, a simulated dataset with a difference in dose-response between the irradiation groups was used to evaluate the statistical approaches with respect to their applicability, usability and validity.

## 2. Results

The results of the different statistical methods for analysing experimental and simulated tumour-growth data are summarized in Table 1 and Table 2, respectively. 

**Figure 1:**
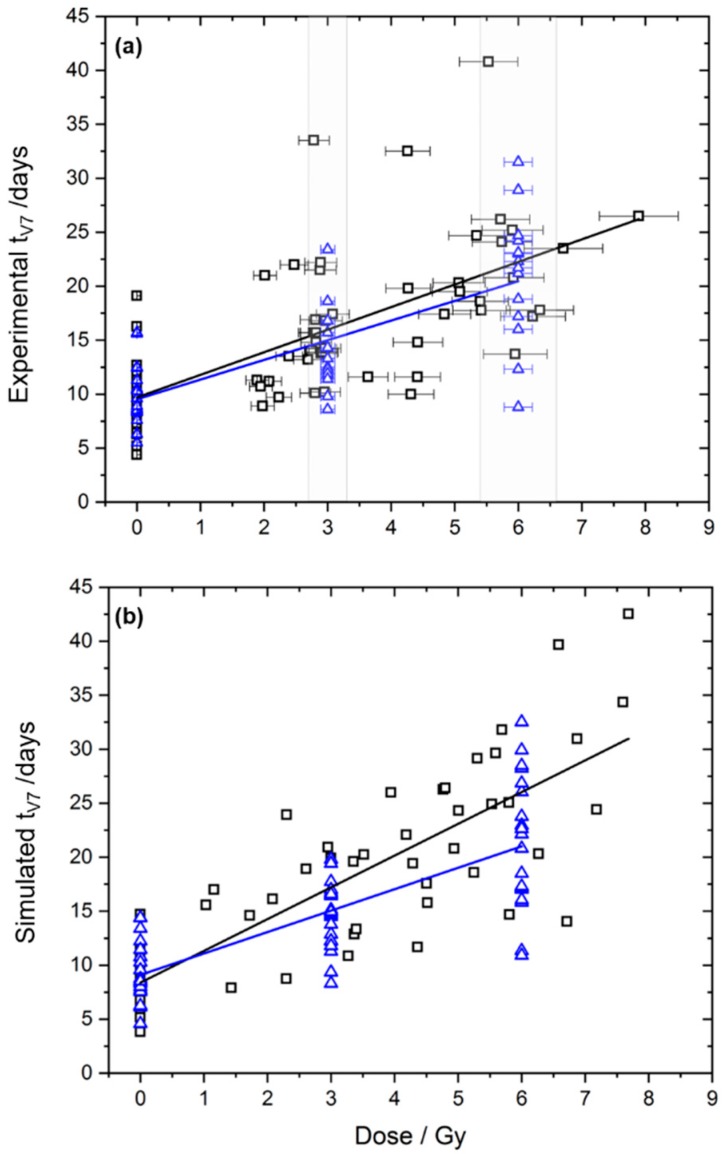
Experimental (**a**) and simulated (**b**) tumour growth data, i.e., time to achieve sevenfold relative volume increase (*t*_V7_), and the corresponding linear regressions for treatment with laser-driven (black squares) and Linac electrons (blue triangles). For the experimental data, the dose region useable for conventional analysis is marked in grey. Therefore, black squares outside the grey area mark mice, which were not included in the conventional analysis.

For the experimental data, no significant differences between the beam qualities were observed by any of the statistical methods (Table 1), as reported previously [12]. The tumour-growth time to reach the sevenfold volume (*t*_V7_) in the 3 Gy group was somewhat longer after irradiation with laser-driven electrons (mean 16.46 days) than after irradiation with the Linac (mean 13.88 days). However, this difference was not statistically significant, as estimated by the conventional method (*p* = 0.14) and by the Monte-Carlo-based method (*p* = 0.18). Figure 1a shows the experimental data and the corresponding linear regression lines, which are close to each other.

In addition to the experimental data, a simulated dataset was generated, in which the laser-driven irradiation led to a dose-response slope that was 50% larger than that of the Linac (Figure 1b). Results of the statistical tests are presented in Table 2. The difference in the slope of the dose response between the groups was not detected using the conventional method (*p* = 0.15). All other statistical methods, however, were able to identify this difference. The Monte-Carlo-based method identified a difference in the slope between 0 Gy and 3 Gy (*p* = 0.012). In linear regression, the differing dose response was reflected by the significant interaction term *b*_DoseGroup_ (*p* = 0.006) and by the overall R² test (*p* = 0.001). Similar results were obtained using Cox regression, where the differing dose-response relationship was reflected by the interaction term *β*_DoseGroup_ that was significantly different from zero (*p* = 0.049) and by the overall likelihood-ratio test (*p* = 0.010).

The presented simulation was repeated 10000 times with different randomly chosen dose and *t*_V7_ values. Overall, the power to detect the existing difference between the groups was only 42% for the conventional method, while the Monte-Carlo-based method reached a higher power of 75%, linear regression achieved a power of 93% and Cox regression of 87%, Table 2.

## 3. Discussion

The starting point for the statistical analyses performed in this manuscript was the substantial exclusion rate of animals from the analysis of a treatment comparison study [12] due to deviations from the prescribed dose. Following the translational chain from bench to bedside, the radiobiological effectivity of laser-driven electrons and conventional Linac electrons was compared to reveal potential pitfalls of the new acceleration regime. Although the campaign itself was performed successfully, 43% of all animals treated with laser-driven electrons had to be excluded from analysis due to deviations of more than 10% from the prescribed radiation dose. This lowers the statistical power to reveal a significant difference between the beam qualities. 

Conventionally, growth data from xenograft subcutaneous tumours were obtained for dedicated, pre-defined treatment groups [13,14,15,16,17] with a sufficient number of animals. Since the allocation in different groups took place before treatment, deviations from the treatment schedule and censoring of animals during follow-up must be taken into account. The latter is considered both in planning of an animal trial and in analysis using approaches that allow for handling censored tumour growth data [13,18,19]. At conventional accelerators, like X-ray tubes or clinical Linacs, deviations from the scheduled treatment regime are very rare. Hence, there was no standard approach available that can handle data with substantial dose deviations as occur, e.g., at the experimental laser-driven accelerator considered here. In a similar experiment with pulsed proton beams, Zlobinskaya et al. [20] circumvented the grouping problem by analysing the growth time for each of the treated animals individually. 

To improve the analysis of animal trials at experimental radiation sources, in this manuscript, different statistical approaches were compared based on the data published by Oppelt et al. [12]. We considered the conventional method, including only animals with an applied dose close to the prescribed dose and as alternatives a Monte-Carlo-based method, linear regression and Cox regression, which allow for including animals with applied doses that strongly deviate from the prescribed dose. As for the previous publication of Oppelt et al. [12] no significant differences between Linac and laser-accelerated electron treatment were obtained for the time to sevenfold tumour growth (*t*_V7_) regardless the method applied. Also for other endpoints, i.e., *t*_V3_, *t*_V5_ and *t*_V10_, a dose response similar to Figure 1a with no significant difference between the radiation sources was observed and the variability between individual mice was similar to *t*_V7_. The large variability of the tumour growth data (Figure 1a) complicates the comparison of the two treatment regimens and the detection of significant differences between the radiation modalities. With respect to this large variability, one may question the previously applied threshold of the conventional method excluding animals with more than 10% deviation from the prescribed dose. Increasing this threshold would improve the power of the conventional method, while the precise assignment of mice to particular dose groups would be lost. 

Compared to patient treatment with large inter-patient variability, in preclinical experiments aiming on the comparison of treatment regimes, one tries to minimize the variability as much as possible. For the experiment described in Oppelt et al. [12], a previously established protocol was applied, which used mice of a strain with defined immune status, age and radiation doses, showing measurable differences between dose groups. However, despite standardized handling procedures, subtle changes, i.e., in the number of tumour cells inoculated in the mice [12], in the position of inoculation, in the stress status of the individual mouse etc., might result in variations in the tumour radiation response, as visible in Figure 1a. This biological variability is hard to predict and must be taken into account by a sufficient number of animals per group and improved tumour models [16]. 

In order to reveal advantages of the applied statistical methods, a dataset was simulated in which laser-driven electrons deliberately led to a steeper dose-response curve than Linac electrons. Overall, the conventional method, excluding animals with doses too far from the prescribed dose, was able to detect the differing dose response only with a probability of 42%, while the other methods showed a power of more than 75%. The highest power was reached by linear regression, which is due to the assumed linear dose-response relationship in the simulation. It is expected that for a non-linear dose response, the power of linear regression would decrease, while the power of the nonparametric Monte-Carlo-based method would remain stable.

The considered statistical methods have different advantages and disadvantages: The conventional method applies *t*-tests for every dose level. In addition to the reduced power due to large drop-out, repeated testing generates a multiple testing problem and applying corresponding corrections would further reduce the power. However, the conventional method does not use a specific assumption on the dose-response relationship, as is required for the regression approaches. The developed Monte-Carlo-based method is similar to the conventional method. It includes all data, does not assume a dose-response relationship and analyses the change in response in neighbouring dose groups. However, in contrast to the other methods, it is not available in standard statistical software and first has to be implemented. While linear regression assumes a linear dose response, Cox regression models the hazard function of tumour recurrence that is related to the tumour control probability using a linear dose dependency. It is also able to handle animals that do not reach the considered endpoint as censored observations, thereby further increasing the sample size. More advanced models like linear mixed models or time-dependent Cox regression also with non-linear terms may be considered as further alternatives [21].

## 4. Materials and Methods 

### 4.1. Input Data Sets

#### 4.1.1. Experimental Data

In the experiment described by Oppelt et al. [12], tumour-bearing mice were irradiated with a prescribed dose of 0, 3 or 6 Gy. After electron treatment, either with laser-driven or conventional Linac electrons, the tumour growth was followed up to a predefined final size. The tumour growth time was determined by the time needed to develop a tumour size, which is a multiple of the size at irradiation. In this manuscript, the time for observing a sevenfold volume, *t*_V7_, was considered and analysed by different statistical approaches. 

Animal numbers are summarised in Table 3. There were 47 mice irradiated at the laser accelerator and 27 mice irradiated at the Linac with a prescribed dose of 3 Gy or 6 Gy. Additionally, 41 mice at the laser accelerator and 20 mice at the Linac were used as controls (0 Gy), which were not irradiated but handled in the same way. In Oppelt et al. [12], 20 of the 47 mice irradiated at the laser accelerator were excluded (43%). The detailed experimental data, comprising treatment doses and the corresponding tumour growth data for the two radiation qualities are tabularized in the Supplement (Tables S1 and S2).

#### 4.1.2. Simulated Data

In the experimental data, no difference between laser-driven and conventional Linac electrons was observed. Thus, to reveal advantages of the applied statistical methods, we simulated dose-response data in which we deliberately included a different dose-response relationship for both groups. For simulating the laser-irradiated mice, 40 data points were generated in three steps. First, a dose *D* was chosen as uniformly distributed in the interval [1,8] Gy. The tumour growth time was then estimated by a normal distribution with mean μ and standard deviation σ according to

μ = 9 + 3 × *D* (1)

and

σ = 0.28 × μ(2)

The given parameters of Equations (1) and (2) were chosen such that the simulated data distribution resembled the experimental data. For 20 control animals, the dose 0 Gy was used.

The process of generating the simulated data for the mice irradiated by the Linac was similar. It differed in the distribution of dose *D*, using the fixed dose values of 0 Gy, 3 Gy and 6 Gy, including 20 data points for every dose group. Furthermore, the slope of the dose response was reduced, i.e., Equation (1) was replaced by:

μ = 9 + 2 × *D* (3)

Results of the statistical methods are presented for one particular example in Table 2. Furthermore, the outlined procedure was repeated 10000 times using different random realizations. For every statistical method, the power to detect the difference in the dose-response relationship was calculated as the ratio of the number of significant results divided by 10000. Methods with a high power should be preferred.

### 4.2. Methods for Analysing Tumour Growth Data

The statistical tests described in the following were performed using SPSS Statistics version 25 (IBM Corporation, Armonk, NY, USA), while Monte-Carlo sampling was performed in Python (Python Software Foundation, Python Language Reference, version 3.6). In this manuscript, *p*-values smaller than 0.05 were considered as statistically significant.

#### 4.2.1. Conventional Analysis

For calculation of the mean tumour growth time per dose group, animals with applied doses not too far from the prescribed dose were included, leading to the 0 Gy, 3 Gy and 6 Gy dose groups. Tumours with more than 10% deviation in the absolute dose were excluded from analysis, i.e., the dose had to be within the intervals of [2.7; 3.3] Gy or [5.4; 6.6] Gy, respectively. After that selection, the mean *t*_V7_ of the selected *N* mice and its standard deviation were calculated for every dose group. The data of the two beam qualities were then compared by a two-sided *t*-test with Welch correction. 

#### 4.2.2. Monte-Carlo-Based Method

**Figure 2:**
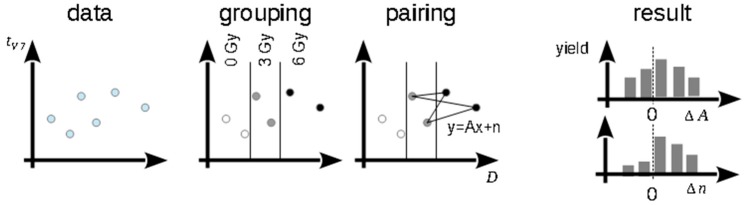
Scheme of the Monte-Carlo-based method. *A*: slope, *n*: intercept.

In contrast to the conventional analysis, the Monte-Carlo-based method includes all irradiated animals, but it does not require the explicit specification of a regression function as the following alternatives methods. The Monte-Carlo-based method is visualized in Figure 2. Data points (*D*_i_, *t*_V7,i_) for each beam quality were divided into three groups according to the applied dose, named “0 Gy”, “3 Gy” and “6 Gy”. In general, every combination of two data points belonging to two different dose groups defines a linear function with a slope parameter *A* and a constant *n.* We randomly selected as many pairs as there were data points in the dose group with the smallest sample size and calculated *A* and *n* for every pair. Subsequently we calculated the difference of the mean slopes, Δ*A* = $\bar{A}$_Linac_ − $\bar{A}$_Laser_, and constants, Δ*n* = $\bar{n}$_Linac_ − $\bar{n}$_Laser_, between the beam qualities. This bootstrap procedure was repeated 10000 times for pairs based on data points from the 0 Gy and 3 Gy groups and for pairs based on data from the 3 Gy and 6 Gy groups. A significant difference in slope or constant between the beam qualities was observed if the central 95% range of the corresponding distributions did not include the value 0. To increase robustness, pairs with too small dose differences were excluded using a minimally allowed difference of 0.5 Gy. 

The grouping of the experimental data from the Linac as well as of the simulated Linac data, was based on the prescribed dose, which was close to the applied dose (0 Gy, 3 Gy or 6 Gy). For the experimental laser-driven electron data, the dose was prescribed accordingly (0 Gy, 3 Gy and 6 Gy), but may differ from the applied dose, which is used for the calculation. Here the groups were defined by the intervals of [1.0; 4.5) Gy and [4.5; 8.0] Gy containing 26 and 21 data points, respectively. This definition was also used for the simulated laser-driven irradiations. 

#### 4.2.3. Linear Regression

This method is based on the assumption of a linear dependency of the tumour-growth time on the applied dose, which was observed, e.g., in an independent experiment with the same tumour model [16] using irradiation with 200 kV X-rays. The high-energy electrons may exhibit the same dose-effect relationship, because it is also a beam quality with low linear energy transfer.

Linear regression was performed using

*t*_V7_(*D*) = *b*_Dose_*D* + *b*_0_ (4)

where *t*_V7_(*D*) are the *t*_V7_ derived for individual tumours treated with a certain dose *D*, and the parameters *b*_0_ and *b_Dose_* were fit to the data. The fit was performed for both beam qualities individually, including all irradiated animals, as shown in Figure 1. The quality of the fit can be measured by the coefficient of determination *R*^2^.

To compare the slopes and intercepts of the regression lines between the radiation qualities, the following multivariable model was applied,

*t*_V7_(*D*) = *b*_Dose_*D* + *b*_Group_*G* + *b*_DoseGroup_*DG* + *b*_0_ (5)

where the group variable *G* represents the radiation quality and *b* are the fit coefficients. Here, we set *G* = 0 for tumours treated with laser-accelerated electrons and *G* = 1 for those treated with electrons delivered by a clinical Linac. The comparison of the two beam qualities was evaluated by the *p*-values corresponding to *b*_Group_ and *b*_DoseGroup_. If *b*_Group_ significantly differs from 0, a global shift between the growth times of both radiation qualities is observed, while a parameter *b*_DoseGroup_ significantly different from 0 describes differing slopes of the dose-effect curves. As an alternative, the change in *R²* between a model with *b*_Group_ = *b*_DoseGroup_ = 0 and a model including all fit parameters can be tested for a significant difference from 0 using an F-test.

#### 4.2.4. Cox Regression

Cox regression can handle censored data, i.e., include mice that did not reach the endpoint of achieving a sevenfold volume increase. First, the hazard function given by:

*h*(*t*) = *h*_0_(*t*) exp(*β*_Dose_*D*) (6)

was fitted for the two beam qualities individually. It consists of a time-dependent baseline hazard *h*_0_(*t*) that is not estimated, and a time-independent factor containing the regression coefficient *β*_Dose._ In a second step, multivariable Cox regression was performed by:

*h*(*t*) = *h*_0_(*t*) exp(*β*_Dose_*D* + *β*_Group_*G* + *β*_DoseGroup_*GD*) (7)

where *G* is the same group parameter as used for the multivariable linear regression. A global difference between the groups is obtained if *β*_Group_ significantly differs from zero, while a significant parameter *β*_DoseGroup_ describes a dose-group interaction. As an alternative, a likelihood-ratio test can be performed using the difference in twice the negative log-likelihood between the model with *β*_Group_ = *β*_DoseGroup_ = 0 and the model including all fit parameters. 

## 5. Conclusions

In this work, we applied different statistical approaches to identify differences between the time to sevenfold tumour volume increase after treatment of mice with laser-driven and conventional Linac electrons. Except for the conventional approach, these methods allow for including animals with applied doses considerably differing from the prescribed dose. Overall, a Monte-Carlo-based method, linear regression and Cox regression were more sensitive than the previously used conventional method and may be applied in future studies. 

## Figures and Tables

**Table 1:** Summary of relevant parameters returned by the different statistical methods applied to the experimentally determined tumour growth data in dependence on dose (*D*). Units (days, Gy) are ignored for clarity.

Parameter	Laser-Driven Electrons				Linac Electrons				*p* Value
**Conventional Analysis**									
	**mean**	**sd**		***N***	**mean**	**sd**		***N***	
*t*_V7_(0 Gy)	9.66	2.87		41	9.90	2.64		20	0.75
*t*_V7_(3 Gy)	16.46	5.41		17	13.88	3.97		13	0.14
*t*_V7_(6 Gy)	22.22	7.64		10	20.98	6.05		14	0.68
**Monte-Carlo-Based Method**									
	**Mean**		**sd**		**Mean**		**sd**		***p* Value**
$\bar{n}$ (0 Gy vs. 3 Gy)	9.67		0.52		9.89		0.71		0.83
$\bar{A}$ (0 Gy vs. 3 Gy)	2.13		0.41		1.33		0.42		0.18
$\bar{n}$ (3 Gy vs. 6 Gy)	9.39		4.95		6.75		2.66		0.63
$\bar{A}$ (3 Gy vs. 6 Gy)	2.24		1.04		2.38		0.65		0.90
**Linear Regression**									
	**Value**		**sd**		**Value**		**sd**		
*b*_Dose_	2.09		0.23		1.82		0.25		
*b*_0_	9.70		0.69		9.53		0.90		
	**value**				**sd**				
*b*_Dose_	2.09				0.22				
*b*_Group_	−0.17				1.17				0.89
*b*_DoseGroup_	−0.27				0.34				0.43
*b*_0_	9.70				0.67				
Δ*R*^2^	0.006								0.46
**Cox Regression**									
	**Value**		**sd**		**Value**		**sd**		
*β*_Dose_	−0.43		0.063		−0.44		0.083		
	**Value**				**sd**				
*β*_Dose_	−0.45				0.060				
*β*_Group_	0.12				0.26				0.66
*β*_DoseGroup_	0.053				0.078				0.50
Δ2*log-likelihood	2.08								0.35

Conventional analysis: *t*_V7_(*D*): Time required to observe sevenfold tumour volume increase (days), sd: Standard deviation, *N*: Number of animals; Monte-Carlo-based method: $\bar{n}$, $\bar{A}$: mean intercept and slope (*t*_V7_ *= AD + n*) of randomly selected pairs of *t*_V7_ values of neighbouring dose groups; linear regression: top: Univariable regression using dose (*b*_Dose_) and intercept (*b*_0_) for each irradiation technique, bottom: Multivariable regression including dose (*b*_Dose_), irradiation group (*b*_Group_), their interaction term (*b*_DoseGroup_) and an intercept (*b*_0_) for the combined dataset; Cox regression: Top: Including dose (*β*_Dose_) for each irradiation technique and bottom: Including dose (*β*_Dose_), irradiation group (*β*_Group_) and their interaction term (*β*_DoseGroup_) for the combined dataset.

**Table 2:** Summary of relevant parameters returned by the different statistical methods applied to the simulated tumour-growth data. Units (days, Gy) are ignored for clarity. Significant *p*-values are marked in bold. The power was estimated based on 10000 repetitions of the simulation.

Parameter	Laser-Driven Electrons				Linac Electrons				*p*-Value	Power
**Conventional Analysis**										
	**Value**	**sd**		***N***	**Value**	**sd**		***N***		
*t*_V7_ (0 Gy)	8.31	2.50		20	9.38	2.69		20	0.20	
*t*_V7_ (3 Gy)	17.04	4.55		4	14.49	3.07		20	0.35	
*t*_V7_ (6 Gy)	26.58	8.10		7	21.28	6.09		20	0.15	0.42
**Monte-Carlo-Based Method**										
	**Mean**		**sd**		**Mean**		**sd**			
$\bar{n}$ (0 Gy vs. 3 Gy)	8.31		0.54		9.38		0.59		0.17	
$\bar{A}$ (0 Gy vs. 3 Gy)	3.26		0.54		1.70		0.30		**0.008**	
$\bar{n}$ (3 Gy vs. 6 Gy)	7.65		3.72		7.68		1.86		0.99	
$\bar{A}$ (3 Gy vs. 6 Gy)	3.03		0.83		2.26		0.49		0.44	0.75
**Linear Regression**										
	**Value**		**sd**		**Value**		**sd**			
*b*_Dose_	2.94		0.26		1.98		0.22			
*b*_0_	8.38		0.99		9.10		0.86			
	**Value**				**sd**					
*b*_Dose_	2.94				0.24					
*b*_Group_	0.72				1.32				0.59	
*b*_DoseGroup_	−0.96				0.34				**0.006**	
*b*_0_	8.38				0.92					
Δ*R*^2^	0.041								**0.001**	0.93
**Cox Regression**										
	**Value**		**sd**		**Value**		**sd**			
*β*_Dose_	−0.71		0.10		−0.60		0.089			
	**Value**				**sd**					
*β*_Dose_	−0.73				0.080					
*β*_Group_	−0.04				0.30				0.99	
*β*_DoseGroup_	0.16				0.080				**0.049**	
Δ2*log-likelihood	9.25								**0.010**	0.87

Conventional analysis: *t*_V7_(*D*): Time required to observe sevenfold tumour volume increase (days), sd: Standard deviation, *N*: Number of animals; Monte-Carlo-based method: $\bar{n}$, $\bar{A}$: mean intercept and slope (*t*_V7_ = *AD* + *n*) of randomly selected pairs of *t*_V7_ values of neighbouring dose groups; linear regression: Top: Univariable regression using dose (*b*_Dose_) and intercept (*b*_0_) for each irradiation technique, bottom: multivariable regression including dose (*b*_Dose_), irradiation group (b_Group_), their interaction term (*b*_DoseGroup_) and an intercept (*b*_0_) for the combined dataset; Cox regression: Top: Including dose (*β*_Dose_) for each irradiation technique, and bottom: Including dose (*β*_Dose_), irradiation group (*β*_Group_) and their interaction term (*β*_DoseGroup_) for the combined dataset.

**Table 3:** Overview of the animals allocated and finally analysed for the electron irradiation experiments described in Oppelt et al. [12] and in this manuscript.

	Laser-Driven Electrons			Linac Electrons		
	0 Gy	3 Gy	6 Gy	0 Gy	3 Gy	6 Gy
Allocated	41	29	18	20	13	14
Out of dose tolerance	-	12	8	-	-	-
Final analysis in [12]	41	17	10	20	13	14
Included in present work	41	29	18	20	13	14

## Supplementary Materials

The following are available online at https://www.mdpi.com/2072-6694/11/9/1281/s1, Table S1 and Table S2.
